# A Role for Reactive Oxygen Species Produced by NADPH Oxidases in the Embryo and Aleurone Cells in Barley Seed Germination

**DOI:** 10.1371/journal.pone.0143173

**Published:** 2015-11-18

**Authors:** Yushi Ishibashi, Shinsuke Kasa, Masatsugu Sakamoto, Nozomi Aoki, Kyohei Kai, Takashi Yuasa, Atsushi Hanada, Shinjiro Yamaguchi, Mari Iwaya-Inoue

**Affiliations:** 1 Crop Science Laboratory, Faculty of Agriculture, Kyushu University, Hakozaki, Higashi-ku, Fukuoka, Japan; 2 RIKEN Plant Science Center, Suehiro-cho, Tsurumi-ku, Yokohama, Kanagawa, Japan; Department of Agriculture and Food Western Australia, AUSTRALIA

## Abstract

Reactive oxygen species (ROS) promote the germination of several seeds, and antioxidants suppress it. However, questions remain regarding the role and production mechanism of ROS in seed germination. Here, we focused on NADPH oxidases, which produce ROS. After imbibition, *NADPH oxidase* mRNAs were expressed in the embryo and in aleurone cells of barley seed; these expression sites were consistent with the sites of ROS production in the seed after imbibition. To clarify the role of NADPH oxidases in barley seed germination, we examined gibberellic acid (GA) / abscisic acid (ABA) metabolism and signaling in barley seeds treated with diphenylene iodonium chloride (DPI), an NADPH oxidase inhibitor. DPI significantly suppressed germination, and suppressed GA biosynthesis and ABA catabolism in embryos. GA, but not ABA, induced NADPH oxidase activity in aleurone cells. Additionally, DPI suppressed the early induction of α-amylase by GA in aleurone cells. These results suggest that ROS produced by NADPH oxidases promote GA biosynthesis in embryos, that GA induces and activates NADPH oxidases in aleurone cells, and that ROS produced by NADPH oxidases induce α-amylase in aleurone cells. We conclude that the ROS generated by NADPH oxidases regulate barley seed germination through GA / ABA metabolism and signaling in embryo and aleurone cells.

## Introduction

Seed germination, a crucial stage in a plant’s life, is complicated by several factors, including plant hormones and environmental factors. Plant hormones such as gibberellins (GAs), abscisic acid (ABA), brassinosteroid and ethylene play key roles in germination [1]. In barley (*Hordeum vulgare*), germination is regulated mainly by GA and ABA. The balance between GA and ABA levels is major regulators of dormancy: GAs promote germination and ABA suppresses it [2]. Other regulators include environmental factors, such as light intensity and low temperatures [3], and several signaling molecules, such as nitric oxide (NO) and reactive oxygen species (ROS) [4–7]. In barley, low temperature, and ROS break seed dormancy and promote germination [8–10]. The relationships between ROS, seed dormancy and germination have been reported in many plants, including barley, *Zinnia elegans*, and sunflower [11–15]. Recently, the breaking of dormancy by ROS was reported in relation to hormone signaling in Arabidopsis and barley [10,16]. Exogenous hydrogen peroxide (H_2_O_2_), an ROS, increased ABA catabolism by enhancing the expression of *CYP707A* genes, which encode ABA 8′-hydroxylases, and enhanced the expression of genes for GA synthesis in dormant Arabidopsis seeds [16]. It enhanced genes for GA synthesis (such as *GA20ox1*) in dormant barley seeds, but did not repress ABA signaling in embryos [10].

There are many reports of the interaction of ROS with plant hormones. In guard cells, ROS are considered to be second messengers in the ABA transduction pathway [17,18]: exogenous ABA increases H_2_O_2_ [19,20], which regulates ion channels, leading to stomatal closure [21]. On the other hand, in barley aleurone cells, ROS was induced by GA but not by ABA [22]. ABA increase the tolerance of aleurone cells to H_2_O_2_ by inducing of catalase activity; in contrast, GA reduce the tolerance to H_2_O_2_ by decreasing of catalase, ascorbate peroxidase, and superoxide dismutase activities [23]. The induction of ROS by GA involves the induction of α-amylase and programmed cell death in barley aleurone cells [22–24]. Thus, we hypothesize that ROS play a major role in GA/ABA signaling in embryo and aleurone cells of barley seeds. However, the production mechanism of ROS was not yet well known.

NADPH oxidases in plasma membrane produce superoxide anions (O_2_^−^) by transferring electrons from cytoplasmic NADPH to oxygen, which subsequently dismutate to H_2_O_2_ and O_2_ [25]. Much work has focused on NADPH oxidase-like enzymes (termed *r*espiratory *b*urst *o*xidase *h*omolog: “rboh”), which function in one of the major enzymatic routes of ROS synthesis in plant cells. The identification and characterization of NADPH oxidases have been reported in several plant species, including rice [26,27], Arabidopsis [28,29], tomato [30], potato [31], tobacco [32], and barley [33,34]. NADPH oxidases play many important roles in signaling and development in plants. For example, in Arabidopsis, *atrbohD* and *atrbohF* single and double mutants have compromised responses to pathogen attack and to ABA in guard cells [35,36]; *atrbohC* mutants have defects in root hair development; and *atrbohD/F* double and *atrbohF* single mutants have reduced ABA inhibition of root elongation [36].

NADPH oxidases also act as key proteins in seed biology. In grass seeds, inhibition of NADPH oxidases delayed germination and root growth, but not coleoptile growth [37]. Alternative splicing of *AtrbohB* could be a general mechanism in after-ripening in Arabidopsis seeds: by altered processing of stored pre-mRNAs, seeds could react quickly to environmental changes [38]. ROS produced by the AtrbohB during after-ripening could act via ABA signaling or post-translational protein modifications. We previously reported that NADPH oxidases regulate α-amylase activity and are involved in germination and seedling growth in barley [9]. However, a detailed analysis of NADPH oxidases in barley seed germination is still required. We therefore focused on the relationship between GA/ABA metabolism in embryos, GA/ABA signaling in aleurone cells, and NADPH oxidases during germination, and investigate the role of NADPH oxidases in barley seed germination.

## Materials and Methods

### Plant material

*Hordeum vulgare* L. ‘Himalaya’ grains, which were grown at Kyushu University, were harvested on 5 June 2010. The grains were stored dry at 4°C until the experimental began. Experiments were carried out with non-dormant grains.

### Germination test

Five replications of 20 seeds each were placed on filter paper in a 9-cm Petri dish. Each dish received 6 mL of 0 (distilled water: DW), 0.01, 0.1, 1, or 5 mM diphenylene iodonium chloride (DPI), an NADPH oxidase inhibitor. The dishes were then incubated in the darkness at 22°C, and the germinating seeds, which protruded the radical through the seed coat, was counted daily for 5 days.

### Localization of superoxide anion and hydrogen peroxide in seeds

To examine the localization of superoxide anion (O_2_^−^) and hydrogen peroxide (H_2_O_2_) in seeds, we treated seeds in Petri dishes with DW for 2 days and then incubated hand-cut longitudinal sections in 6 mM nitroblue tetrazolium (NBT) or 4.7 mM 3,3′-diaminobenzidine (DAB) in 10 mM Tris·HCl buffer (pH 7.4) for 30 min. The superoxide anion and H_2_O_2_ were seen as deposits of dark-blue and brown coloration under a stereomicroscope, respectively (Zeiss) [22,39].

### Tissue printing

To examine the localization of *NADPH oxidase* mRNAs in seeds, we performed tissue printing according to the method of Nonogaki et al. [40]. After being soaked for 24 h in water, seeds were longitudinally sliced in two with a razor blade. The cut surfaces were pressed onto a Hybond-N+ membrane for 15 s. The membrane was cross-linked under UV light and hybridized with RNA probes (both sense and antisense). The RNA probes were prepared from PCR products by using NADPH oxidase common primers [9] in a digoxigenin (DIG) labeling kit (Roche Diagnostics). The membrane was prehybridized at 65°C for 1 h in 0.3 M phosphate buffer containing 7% SDS, and then hybridized by incubation in the same buffer with DIG-labeled probes at 65°C for over 15 h. The membrane was then washed in 2× SSC containing 0.1% SDS (15 min), and then in 0.1× SSC containing 0.1% SDS (15 min) at 70°C. It was then blocked with ECL Advance blocking reagent (GE Healthcare) for 1 h and incubated with alkaline phosphatase—conjugated anti-DIG antibody for 1 h at 37°C. Signals were colorimetrically detected by using NBT/BCIP solution (Roche Diagnostics).

### Superoxide anion and hydrogen peroxide contents

Superoxide anion (O_2_^−^) and hydrogen peroxide (H_2_O_2_) contents in embryos isolated after germination treatment or in embryoless half-seeds were measured according to the method of Oracz et al. [13] by using a peroxidase-based assay with 3-dimethylaminobenzoic acid and 1.3 mM 3-methyl-2-benzothiazolinone hydrazone to measure H_2_O_2_ [41] and by examining the oxidation of hydroxylamine to nitrite to measure O_2_^−^ [42].

### Quantitative real-time PCR

Total RNA was extracted from embryos isolated after germination treatment or from embryoless half-seeds by using the SDS/phenol/LiCl method [43]. cDNAs synthesis and the amplification were conducted according to Ishibashi et al. [9]. The amounts of each gene transcript were normalized against the amount of mRNA for *HvActin* [44] by the method of Pfaffl [45]. The sequences for the *HvActin* primer came from Ishibashi et al. [9]; the other primer sequences are shown in S1 Table.

### GA and ABA contents

To measure the GA and ABA contents in embryos, we isolated embryos from 20 dry seeds, 20 seeds that had imbibed for 18 h, and 20 that had imbibed for 30 h and stored them at –80°C. GA levels were measured by means of liquid chromatography—selected reaction monitoring, as described previously [46]. ABA levels were measured by using a Phytodetek Competitive ELISA kit (Agdia) as described in the Phytodetek protocol. Each experiment comprised three biological replicates.

### Enzyme activity

α-Amylase and NADPH oxidase activities were measured according to Ishibashi et al. [9]. α-Amylase activity was measured using Amylase HR Reagent (Megazyme International Ireland). Measurement of NADPH oxidase activity was assayed with reference to the method of van Gestelen et al. [47] and Sarath et al. [37]. Protein concentration was determined according to the Bradford method [48]. The results are expressed as μmol mg^–1^ protein.

## Results

### Inhibition of NADPH oxidases delays barley seed germination

**Fig 1:**
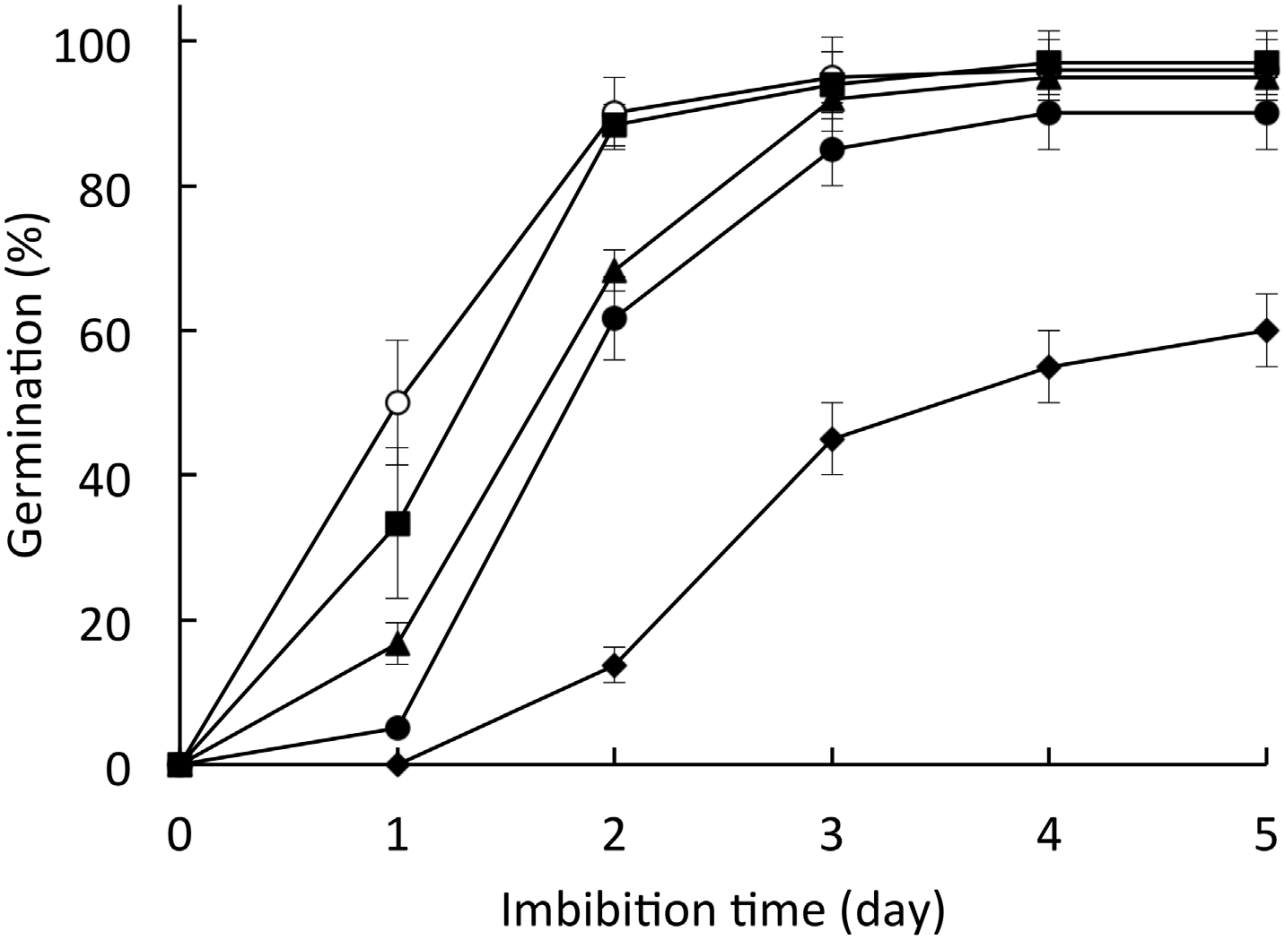
Effect of DPI on barley seed germination. Seeds were incubated in ○ DW, ■ 10 μM, ▲ 100 μM, ● 1 mM, or ◆ 5 mM DPI for 5 days. Values are means ± SD of five replicates.

DW-treated seeds had a germination rate of 50% at 1 day after treatment (DAT), and were fully germinated at 3 DAT. Seeds treated with various concentrations of DPI (shown in parentheses), an NADPH oxidase inhibitor, had germination rates of 35% (10 μM), 17% (100 μM), 5% (1 mM), and 0% (5 mM) at 1 DAT (Fig 1). We defined the mean time required for germination to reach 50% as T_50%_. The T_50%_ values were 1 day (DW), 1.3 days (10 μM DPI), 1.65 days (100 μM DPI), 1.8 days (1 mM DPI), and 3.5 days (5 mM DPI). This result was consistent with previous reports [10, 37]. We used 1 mM DPI in our subsequent analyses according to Bahin et al. [10].

#### NADPH oxidase mRNAs are localized in the embryo and in the aleurone layer of barley seed

**Fig 2:**
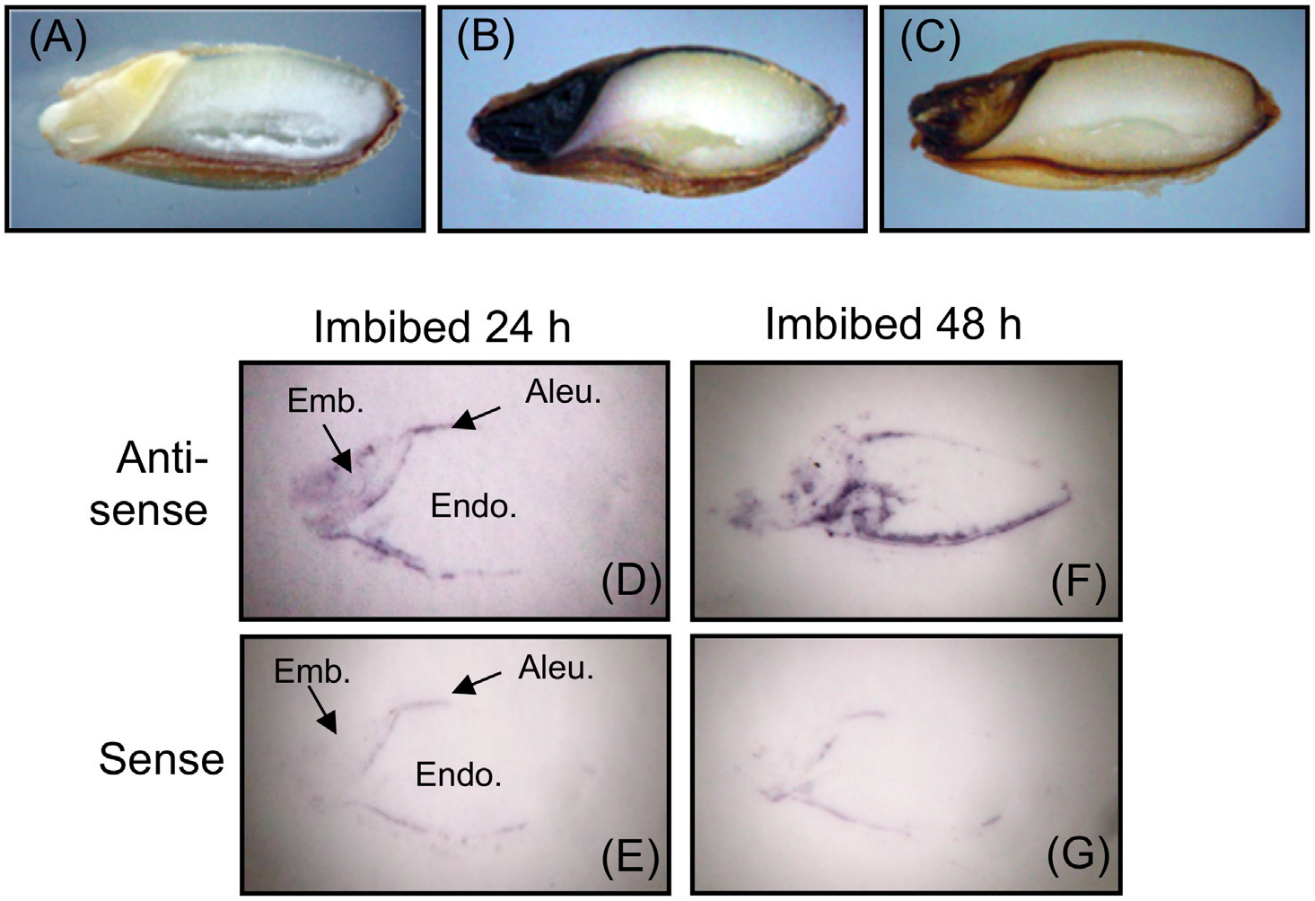
Localization of superoxide anion, hydrogen peroxide, and *NADPH oxidase* mRNA induced after imbibition in barley seeds. (A) Distilled water; (B) NBT; (C) DAB (each for 24 h). (D—G) Expression of *NADPH oxidase* mRNA. Barley seeds imbibed for 24 or 48 h were bisected, and the cut surfaces were printed onto two membranes: (D, F) hybridized with antisense probe for NADPH oxidase; (E, G) hybridized with the corresponding sense probe as a control for non-specific binding. Emb, embryo; Endo, endosperm; Aleu, aleurone layer.

To assay O_2_^−^ and H_2_O_2_ accumulation, we stained barley seeds with NBT and DAB. DW-treated seeds showed clear accumulation of O_2_^−^ and H_2_O_2_ in the embryo and in the aleurone layer, including scutellum epithelial cells, but not in the endosperm (Fig 2B and 2C). We then used tissue printing to determine the localization of NADPH oxidase mRNAs in barley seed during imbibition (Fig 2D–2G). mRNAs were expressed in the embryo and in some parts of the aleurone layer at 24 h, and throughout the embryo and aleurone layer at 48 h (Fig 2D and 2F). These results show that the NADPH oxidases and ROS co-localized.

#### NADPH oxidases regulate GA and ABA contents in embryos during barley seed germination

**Fig 3:**
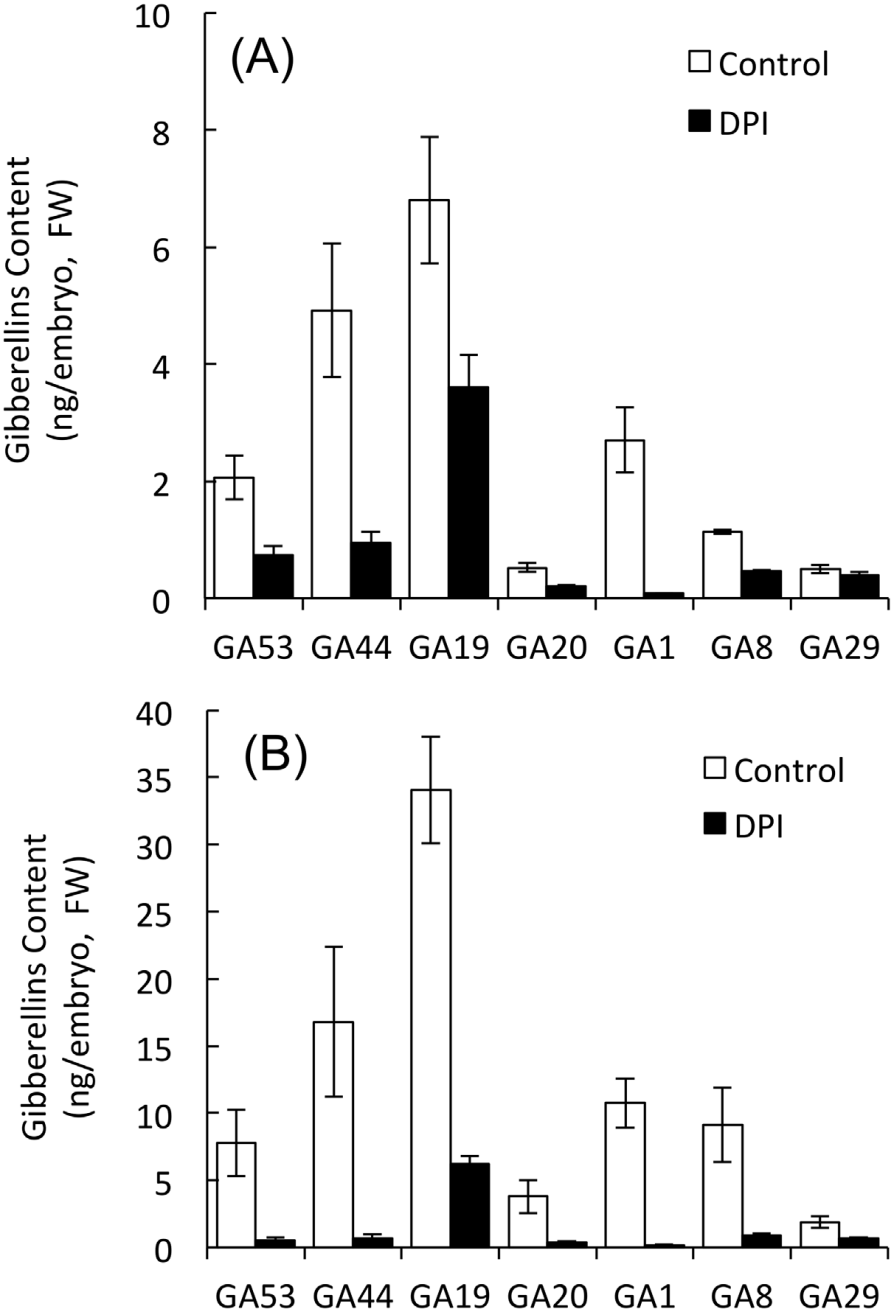
Gibberellin contents in barley seed embryos treated with DPI. Embryos were removed from barley seeds that had been treated with distilled water or 1 mM DPI for (A) 18 and (B) 30 h. Values are means ± SD of five replicates.

**Fig 4:**
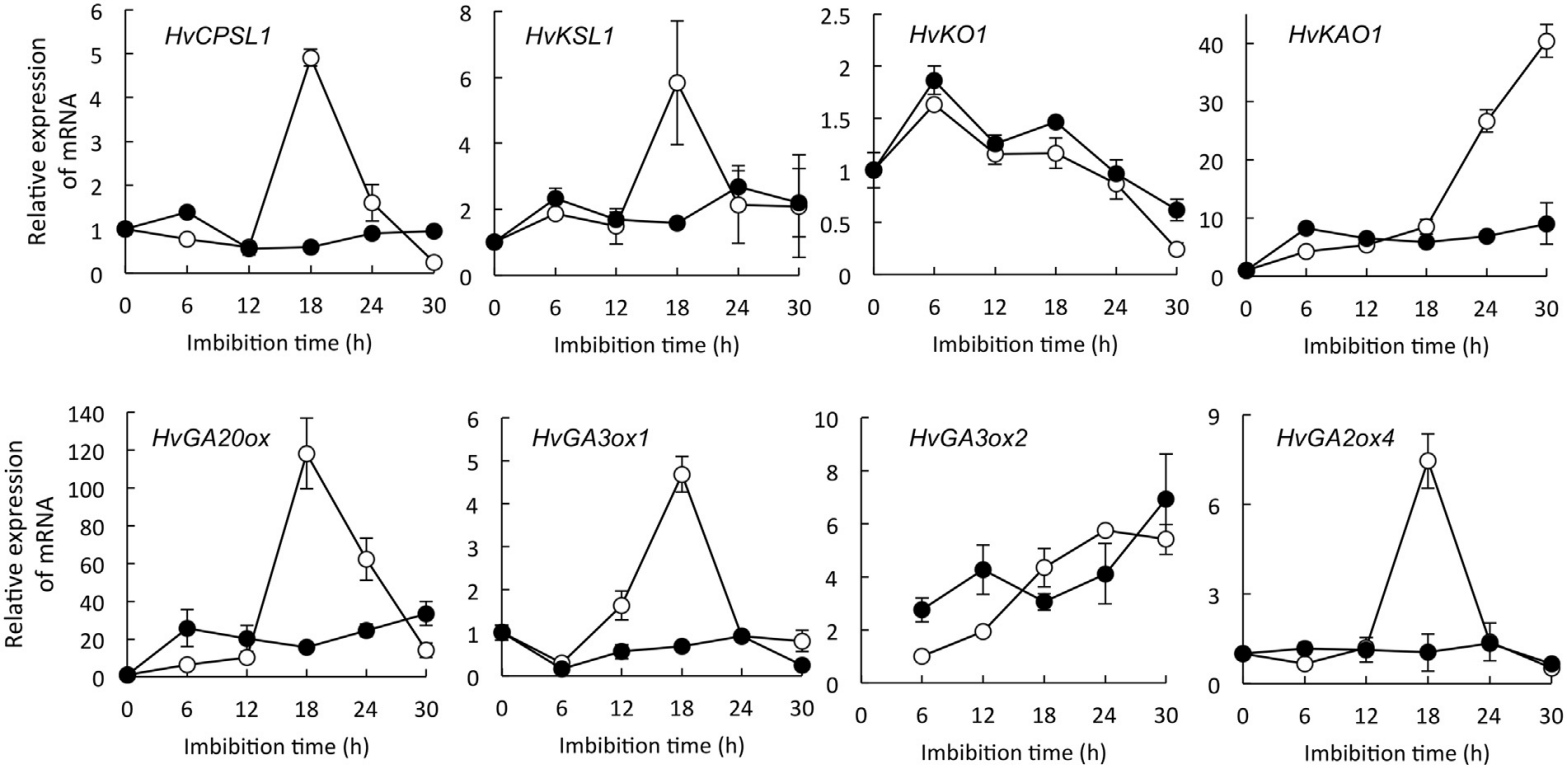
Expression of gibberellin biosynthesis—related genes in barley seed embryos treated with DPI. Embryos were removed from barley seeds that had been treated with ○ distilled water as a control or ● 1 mM DPI. Values are means ± SD of five replicates.

Our data show that NADPH oxidases localized to the embryo (Fig 2). Moreover, the O_2_^−^ and H_2_O_2_ contents of the embryo were suppressed when seeds were treated with DPI (Table 1). To clarify the role of ROS produced by NADPH oxidases in the metabolism of GA and ABA in the embryo, we investigated the expression of genes for GA and ABA biosynthesis and catabolism. At 18 and 30 h after imbibition, the endogenous levels of GA in embryos treated with DPI were much lower than those in embryos treated with DW (Fig 3). GA_1_ (activated gibberellin) levels in embryos treated with DW and DPI were 2.7 and 0.1 ng/embryo (FW) at 18 h, and 10.8 and 0.2 ng/embryo (FW) at 30 h, respectively (Fig 3). The expression of the GA biosynthesis genes *HvGA20ox1* and *HvGA3ox1* and the GA catabolism gene *HvGA2ox4* in DW-treated embryos peaked at 18 h after imbibition, and the expression of *HvGA3ox2* gradually increased after imbibition (Fig 4). However, DPI greatly suppressed the expression of *HvGA20ox1*, *HvGA3ox1*, and *HvGA2ox4*, although *HvGA3ox2* still increased (Fig 4).

**Table 1:** Superoxide and hydrogen peroxide contents in embryos of DPI-treated barley seeds.

	O_2_^−^ (μmol per embryo)		H_2_O_2_ (μmol per embryo)	
	18 h	24 h	18 h	24 h
Control	0.9 ± 0.12	2.08 ± 0.42a	2.88 ± 0.05a	2.26 ± 0.14a
DPI	ND	1.14 ± 0.12b	0.89 ± 0.18b	1.50 ± 0.08b

Embryos (including the scutellum) were removed from barley seeds that had been treated with distilled water or 1 mM DPI for 18 and 24 h. Values followed by the same letter do not differ significantly (*P* < 0.05, Student’s *t*-test). ND, not detected.

**Fig 5:**
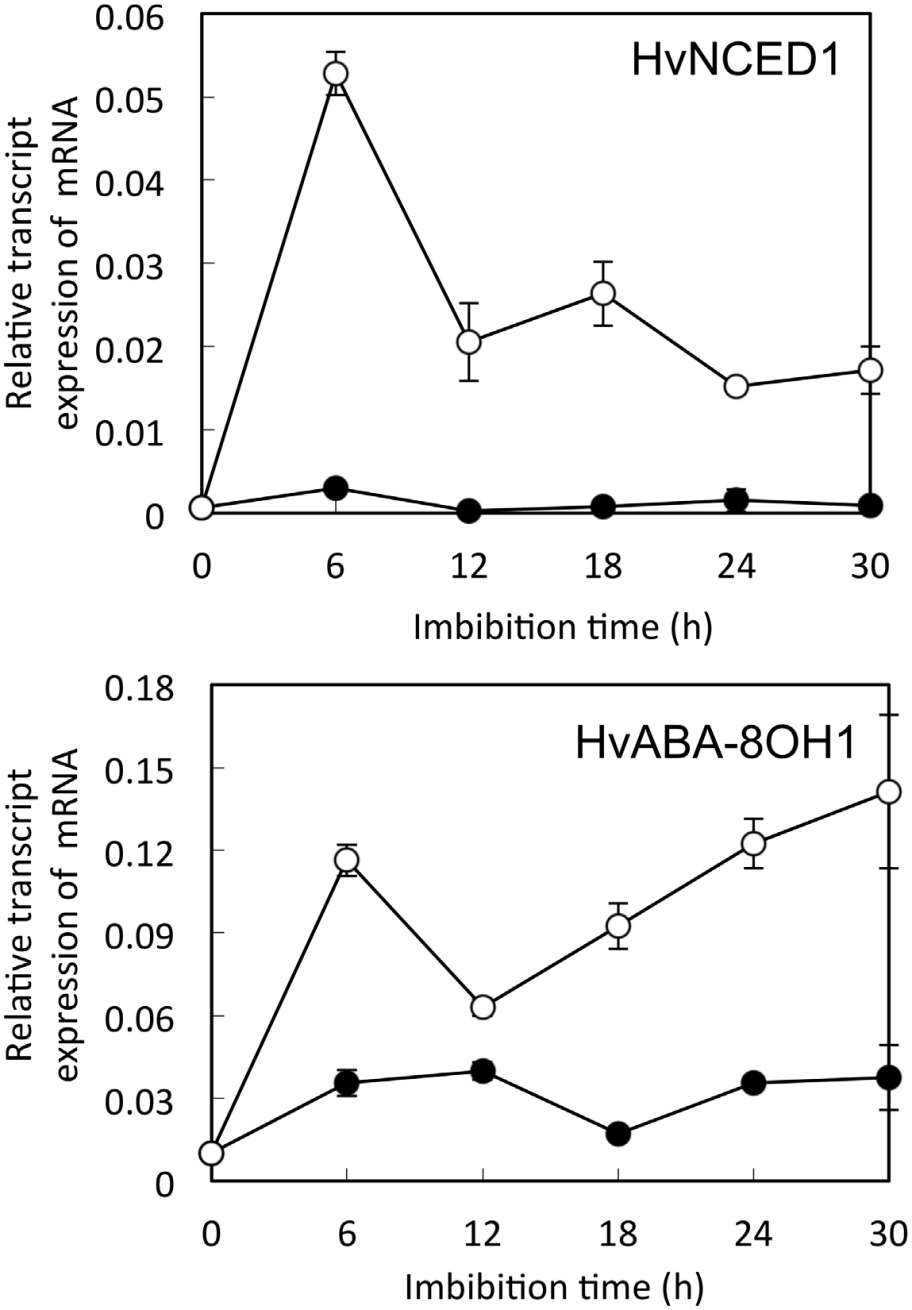
Expression of abscisic acid metabolism—related genes in barley seed embryos treated with DPI. Embryos were removed from barley seeds that had been treated with distilled water or 1 mM DPI. ○ Control, ● DPI treatment. Values are means ± SD of five replicates.

We then measured the accumulation of ABA, which, as an antagonist of GA, inhibits the germination of barley. Endogenous ABA levels were lower at 18 and 30 h after imbibition than in dry seeds. However, they were also lower in the DW-treated embryos than in the DPI-treated embryos (Table 2). Therefore, we examined the expression of genes related to ABA biosynthesis and catabolism. DPI suppressed the expression of *HvNCED1*, a gene involved in ABA biosynthesis, and *HvABA8′OH-1*, a gene required for ABA catabolism (Fig 5).

**Table 2:** ABA content in embryos of DPI-treated barley seeds.

	ABA content (ng per embryo)		
	Dry seeds	18 h	30 h
Control		0.091 ± 0.027a	0.153 ± 0.005a
DPI	6.68 ± 0.36	0.244 ± 0.018b	0.334 ± 0.104b

Embryos were removed from barley seeds that had been treated with distilled water or 1 mM DPI for 18 and 30 h. Values followed by the same letter do not differ significantly (*P* < 0.05, Student’s *t*-test).

#### ABA suppresses the gene expression and activity of NADPH oxidases in the embryo

**Fig 6:**
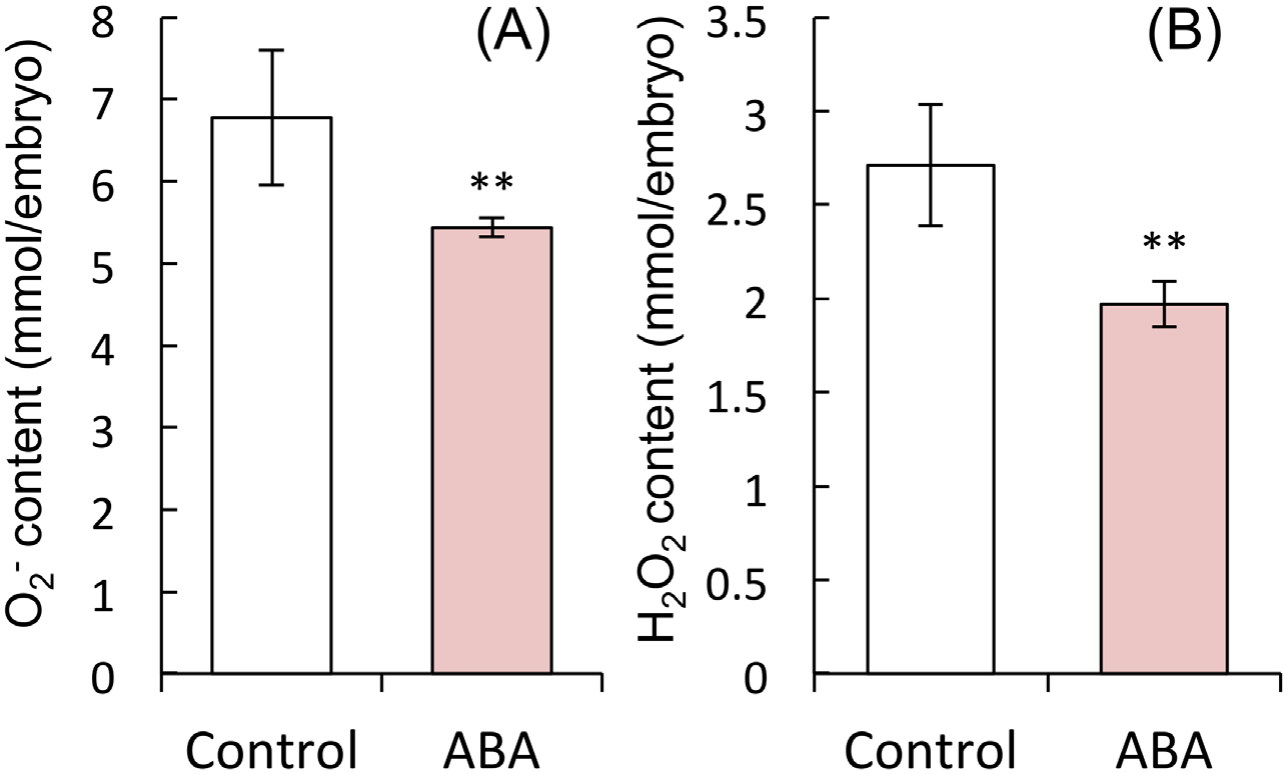
NADPH oxidase activity and ROS content of barley seed embryos treated with ABA. Embryos were removed from barley seeds that had been treated with distilled water or 50 μM ABA for 24 h. (A) O_2_^−^ content; (B) H_2_O_2_ content. (**P < 0.05, Student’s test, *n* = 5).

**Fig 7:**
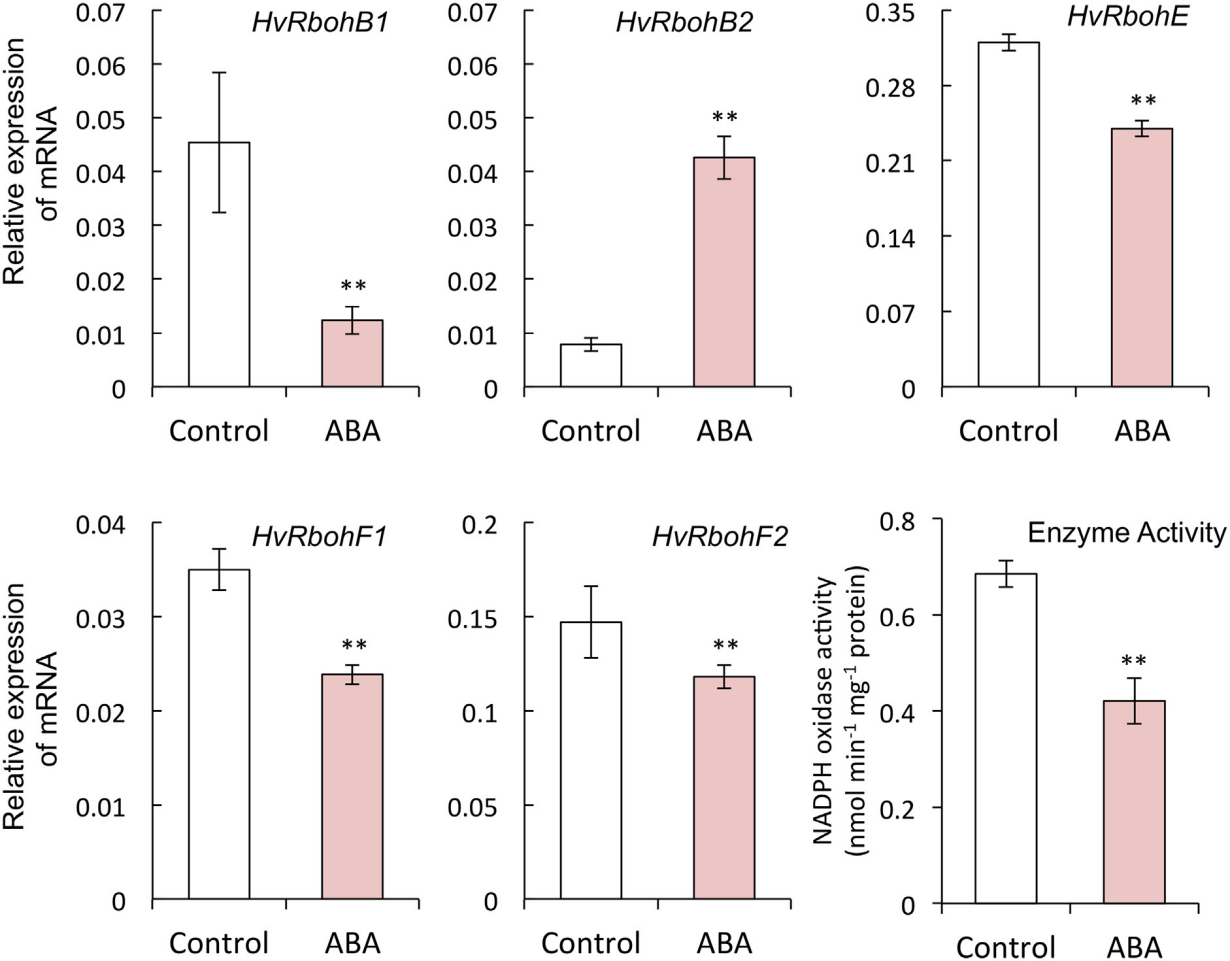
Expression of barley *NADPH oxidase* mRNAs in barley seed embryos treated with ABA. Embryos were removed from barley seeds that had been treated with distilled water or 50 μM ABA for 24 h. (**P < 0.05, Student’s test, *n* = 5).

We examined the effect of ABA on ROS content, gene expression, and the activity of NADPH oxidases in embryos. ABA treatment suppressed O_2_^−^ and H_2_O_2_ content in embryos (Fig 6), as well as the expression of *HvRbohB1*, *HvRbohE*, *HvRbohF1* and *HvRbohF2*, but not *HvRbohB2*, all of which are expressed in barley seeds, and enzyme activity of NADPH oxidase (Fig 7).

#### NADPH oxidases regulate α-amylase activity in aleurone cells

**Fig 8:**
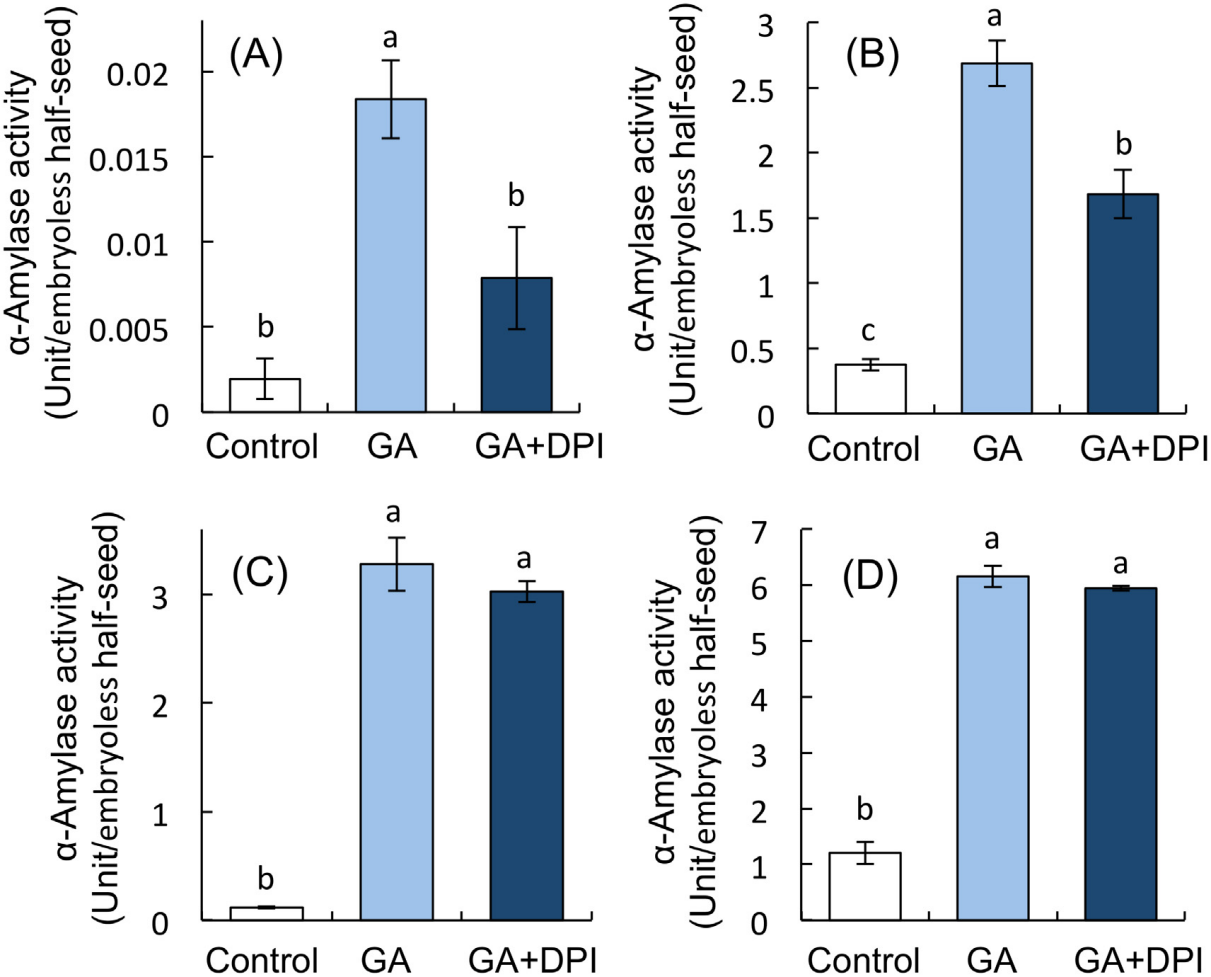
Induction of α-amylase in embryoless half-seeds treated with distilled water, 1 μM GA, or 1 μM GA + 1 mM DPI for (a) 12, (b) 24, (c) 36, and (d) 48 h. Bar with different letters differ significantly (*P* < 0.05, Tukey’s test, *n* = 3).

We previously reported that hydrogen peroxide produced by GA in aleurone cells regulates the induction of α-amylase [22]. In this study also, GA increased superoxide and hydrogen peroxide content in aleurone cells, whereas DPI suppressed superoxide content after 24 h of treatment (Table 3). Therefore, to investigate whether NADPH oxidases contribute to α-amylase activity in aleurone cells, we examined the α-amylase activity in embryoless half-seeds treated with DW, GA, and GA + DPI. GA markedly increased the α-amylase activity in embryoless half-seeds, and DPI suppressed this increase, especially at 12 and 24 h after treatment (Fig 8).

**Table 3:** Superoxide and hydrogen peroxide contents in DPI-treated embryoless half-seeds.

	O_2_^−^ (μmol per half seed)		H_2_O_2_ (μmol per half seed)	
	24 h	36 h	24 h	36 h
Control	4.15 ± 0.30c	6.30 ± 0.33b	2.76 ± 0.19b	0.97 ± 0.26b
GA	10.11 ± 1.74a	12.25 ± 1.61a	5.46 ± 0.19a	3.92 ± 0.10a
GA + DPI	7.20 ± 0.68b	10.38 ± 0.05a	5.99 ± 0.45a	4.25 ± 0.37a

Embryoless half-seeds were treated with distilled water (control), 1 μM GA, or 1 μM GA + 1 mM DPI for 24 and 48 h. Values followed by the same letter do not differ significantly (*P* < 0.05, Tukey’s test).

#### NADPH oxidase activity in aleurone cells is regulated by GA, ABA, and Ca2+

**Fig 9:**
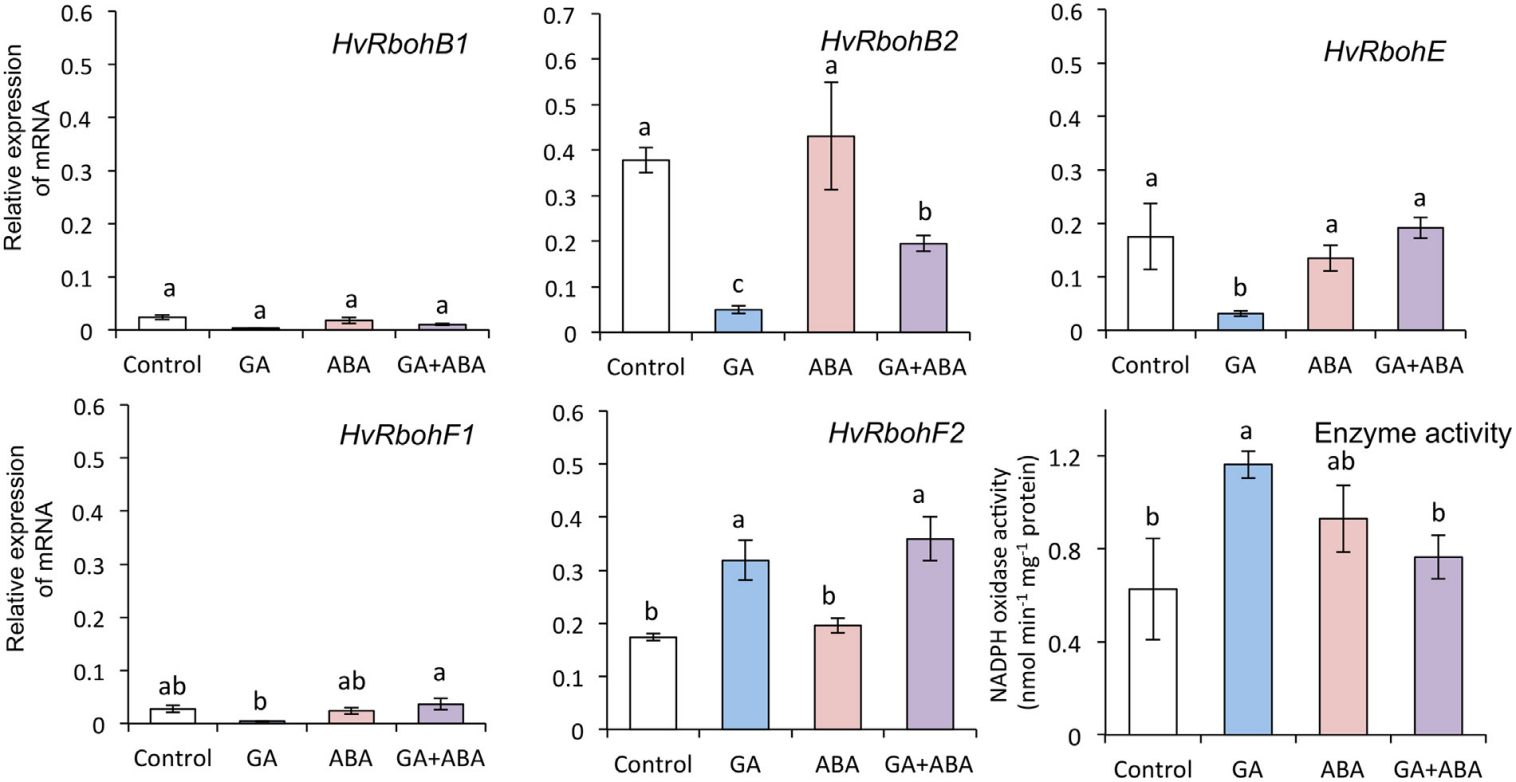
Gene expression and activity of NADPH oxidases in embryoless half-seeds treated with distilled water, 1 μM GA, or 1 μM GA + 50 μM ABA for 24 h. Bar with different letters differ significantly (*P* < 0.05, Tukey’s test, *n* = 3).

To investigate the effect of GA and ABA on NADPH oxidase activity in aleurone cells, we examined the gene expression and activity of NADPH oxidases in embryoless half-seeds treated with GA and ABA. GA increased the expression of *HvRbohF2* mRNA in embryoless half-seeds, but decreased that of other *HvRboh* mRNAs (Fig 9). It doubled the overall activity of NADPH oxidases. ABA suppressed these increases.

**Fig 10:**
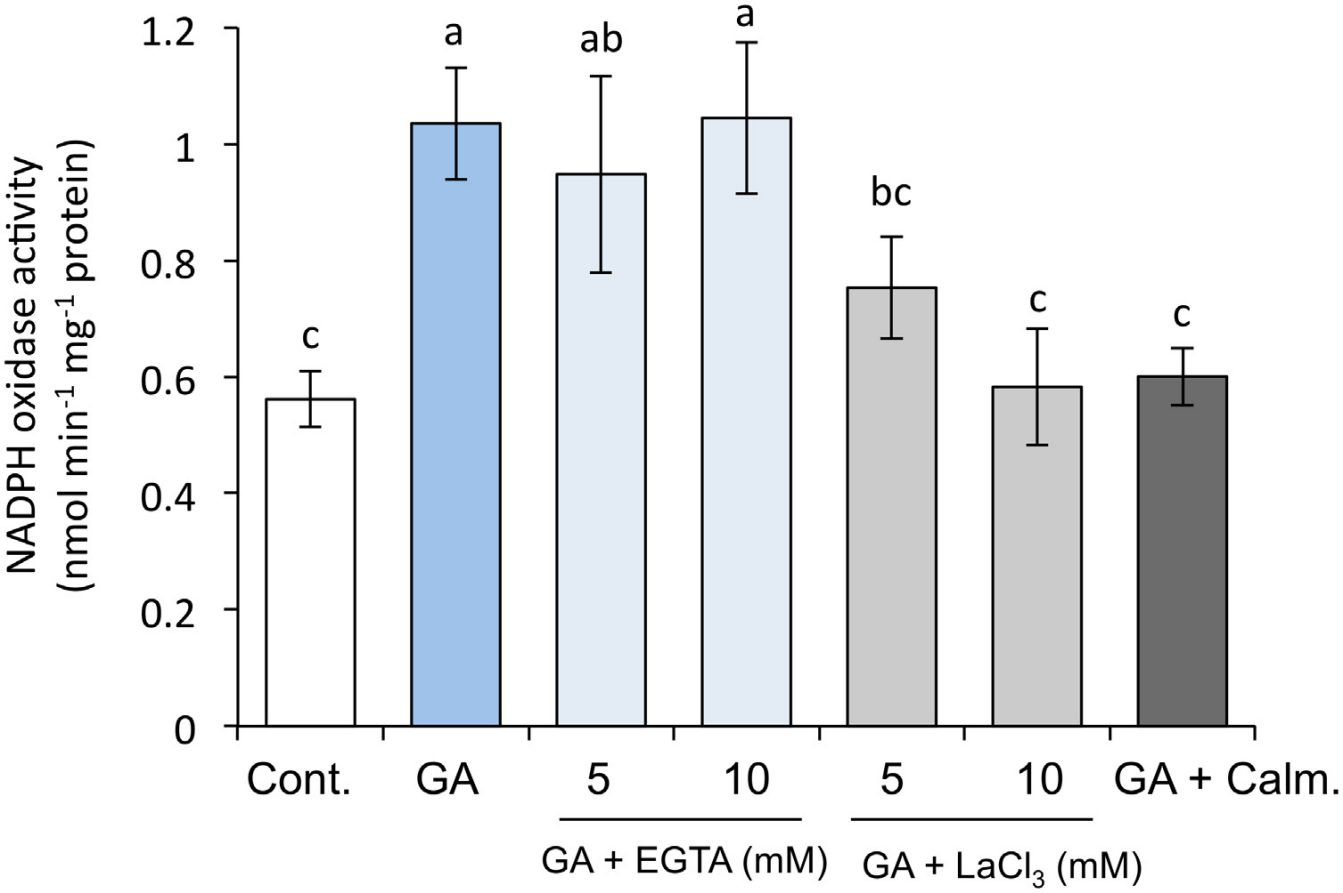
Effect of calcium ions on NADPH oxidase activity in embryoless half-seeds treated with distilled water, 1 μM GA, 1 μM GA + 5 or 10 mM EGTA, 1 μM GA + 5 or 10 mM LaCl_3_, or 1 μM GA + 50 mM calmidazolium for 24 h. Bar with different letters differ significantly (*P* < 0.05, Tukey’s test, *n* = 5).

The NADPH oxidases of plants have an EF-hand motif, which is a calcium-binding site, and are activated by Ca^2+^ [49]. GA increases Ca^2+^ and calmodulin, is the predominant Ca^2+^ sensor, and plays a crucial role in Ca^2+^ signaling in barley aleurone cells; ABA antagonizes these effects of GA [50, 51]. Therefore, we used EGTA (a calcium chelator), La^3+^ (a Ca^2+^ channel blocker), and calmidazolium (a Ca^2+^ and calmodulin inhibitor) to investigate whether GA-induced Ca^2+^ and calmodulin in aleurone cells activate the NADPH oxidases (Fig 10). EGTA had little effect on the NADPH oxidase activity induced by GA; however, La^3+^ markedly suppressed the NADPH oxidase activity in a concentration-dependent manner. Calmidazolium also suppressed the NADPH oxidase activity to the control level.

## Discussion

Our results show that NADPH oxidases perform a crucial function in barley seed germination. The first line of evidence supporting this conclusion is the correspondence between the localization of NADPH oxidase mRNAs and the production of O_2_^−^ and H_2_O_2_ in seeds after imbibition (Fig 2). H_2_O_2_ is produced during the early imbibition period in seeds of soybean [52, 53], maize [54], wheat [55], and *Zinnia elegans* [12]; ROS produced after imbibition are assumed to play a role in seed germination. For instance, the ROS in soybean seed promote seed germination by regulation of ethylene biosynthesis [53]. Here, barley seed germination was suppressed by DPI, an inhibitor of NADPH oxidases, which produce O_2_^−^ (Fig 1). We previously reported that DPI does not suppress gene expression, only the activity of NADPH oxidase in barley seed germination [9], although DPI can inhibit flavin-containing enzymes [56]. The seed germination status in barley is determined by the balance between the actions of GA and ABA [57]. The levels of GA and ABA in germinating seeds are regulated mainly by transcriptional control of the biosynthesis and catabolism genes. We therefore investigated the relationship between the ROS produced by NADPH oxidase and GA/ABA in barley seed germination.

The DPI-induced reduction of ROS in embryos led to the suppression of their GA content (Table 1; Fig 3). H_2_O_2_ enhances the expression of *HvGA20ox1* in the embryos of dormant barley seeds [10], and of *GA3ox* and *GA20ox* in Arabidopsis, whereas DPI suppresses it [16]. In the present study, DPI suppressed the expression of *HvGA20ox1* and *HvGA3ox1* (Fig 4). GA biosynthesis genes are expressed in the epithelium cells that face the endosperm [58]. We confirmed the localization of NADPH oxidase mRNAs in scutellum epithelial cells in barley seeds after imbibition (Fig 2). These results indicate that ROS produced by NADPH oxidase in embryos (especially scutellum epithelial cells) is specifically involved in GA biosynthesis upon seed germination.

ABA inhibits germination and induces seed dormancy [59]. Treatment of pea seeds with H_2_O_2_ decreases the ABA content [60]. In the present study, the ABA content in the embryos of DPI-treated seeds was higher than that in the control (Table 2). DPI markedly suppressed the expression of both *HvNCED1* (a gene involved in ABA biosynthesis) and *HvABA8′OH-1* (involved in ABA catabolism) in embryos (Fig 5). *HvNCED1* plays a key role in regulating ABA levels under conditions that inhibit germination, such as at 30°C in the dark [61] and in the light [62]. In contrast, for dormant barley seeds to germinate, ABA catabolism by ABA8′-OH in the embryo is more important than ABA biosynthesis by NCED [63]. The ROS produced by NADPH oxidases in embryos might therefore be related to the degradation of ABA induced by *HvABA8′OH-1*.

We also examined the relationship between ABA and NADPH oxidase. ABA suppressed the expression of *HvRbohB1*, *HvRbohE*, *HvRbohF1*, and *HvRbohF2*, but not that of *HvRbohB2*, and also suppressed the activity of NADPH oxidases in embryos (Fig 7). Additionally, it suppressed the ROS content in embryos (Fig 6). In plants, NADPH oxidases are involved in ABA signal transduction. ABA treatment induces the expression of *AtrbohD* and *AtrbohF* in Arabidopsis guard cells [34], and enhances the activity of NADPH oxidase in maize leaves [64, 65]. *AtrbohD* and *AtrbohF* mediate ABA-induced ROS production, ABA activation of Ca^2+^-permeable channels, and ABA-induced stomatal closure [34]. Our results suggest that the suppression of NADPH oxidase by ABA decreases the ROS content in the embryo, although this suggestion is inconsistent with other reports.

We previously reported that ROS produced by GA in aleurone cells negatively regulate ABA signals and consequently promote the induction of α-amylase in aleurone cells [22]. DPI treatment of embryoless half-seeds significantly suppressed the α-amylase activities induced by GA at 12 and 24 h but not at 36 and 48 h after imbibition (Fig 8). Additionally, GA induced the production of O_2_^−^ and H_2_O_2_ in aleurone cells, and DPI suppressed the production of O_2_^−^ by GA at 24 h but not at 36 h after imbibition (Table 3). In barley aleurone cells, ROS are produced by GA and suppressed by ABA [22]. We therefore examined the relationship between NADPH oxidases and GA or ABA in aleurone cells. GA promoted the activity of NADPH oxidases and ABA suppressed this activity, although the expression of *HvRboh* mRNAs except for *HvRbohF2* was suppressed by GA (Fig 9). Gilroy [66] reported that GA increases Ca^2+^ and calmodulin levels in barley aleurone cells, that ABA antagonizes this effect, and that the regulation of cytoplasmic Ca^2+^ and calmodulin is important in the secretion of enzymes such as α-amylase by barley aleurone cells. In addition, the activation of NADPH oxidases depends on the influx of Ca^2+^ into the cytoplasm and on the phosphorylation of the N-terminal region of these enzymes by a Ca^2+^-dependent protein kinase, because the N-terminal region contains regulatory elements such as calcium-binding EF-hands and phosphorylation domains [67]. We therefore examined the relationship between Ca^2+^ and NADPH oxidase activity in aleurone cells (Fig 10). EGTA had no effect on the GA-induced increase in NADPH oxidase activity, perhaps because the chelation ability of EGTA is reduced at low pH, which is the condition of GA-treated aleurone cells [68, 69]. In contrast, LaCl_3_ and calmidazolium markedly suppressed the GA-induced increase in NADPH oxidase activity. These results indicate that the Ca^2+^ induced by GA in aleurone cells activates NADPH oxidase. Previous reports have also suggested that an increase in Ca^2+^ is an immediate response of the barley aleurone layer to GA [70]. Thus, activation of NADPH oxidases by Ca^2+^ rather than via GA-induced expression of *HvRboh*s might contribute to the early induction of α-amylase in barley aleurone cells.

**Fig 11:**
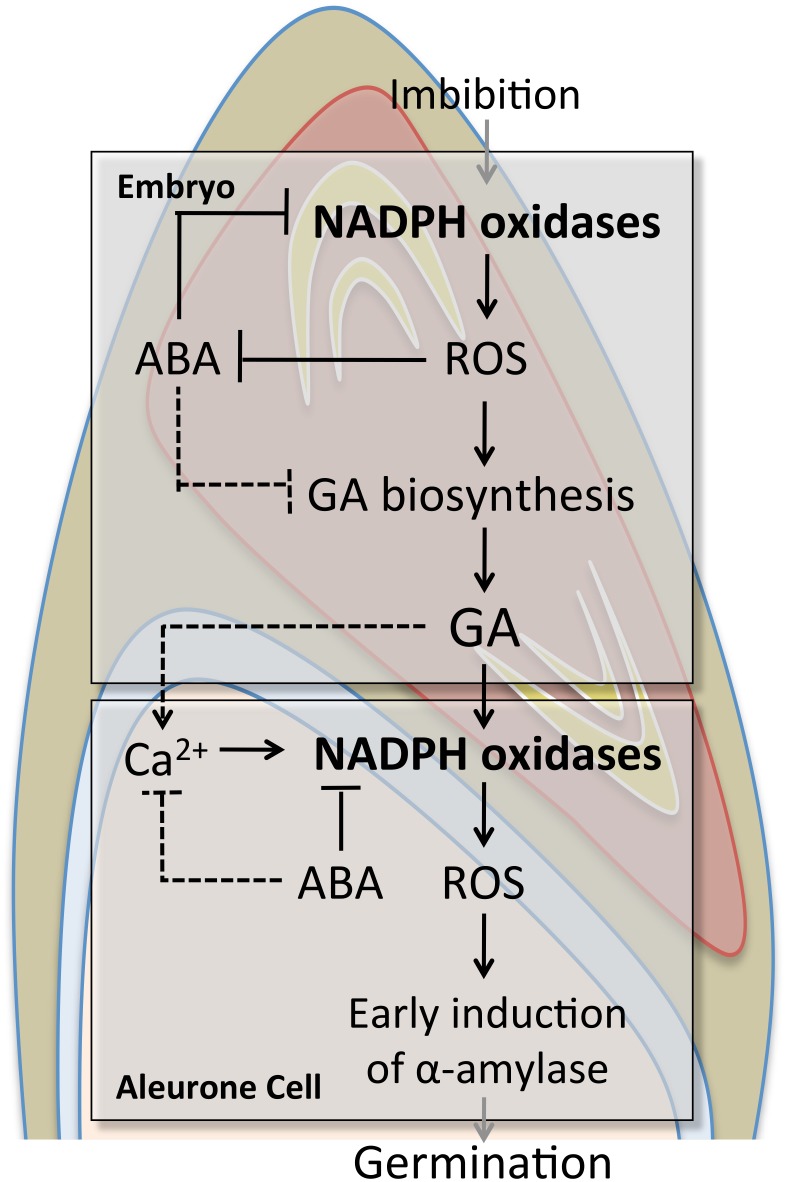
Role of NADPH oxidases in barley seed germination. Black lines, our results; dashed lines, previous reports.

From these results, we propose that NADPH oxidases promote GA biosynthesis and ABA catabolism in germinating barley embryos; that GA induces and activates NADPH oxidases in aleurone cells; and that ROS produced by the NADPH oxidases induce the early induction of α-amylase in aleurone cells. In this way, NADPH oxidases regulate barley seed germination (Fig 11). The data presented here provide new insights into the embryo and aleurone signal pathways involved in barley seed germination and into the roles of NADPH oxidases in these pathways.

## Supporting Information

S1 Table Primer sequences used for real-time PCR. (TIFF)

## Abbreviations

ABA: abscisic acid
ABA8′-OH: ABA8′-hydroxylase
DPI: diphenylene iodonium chloride
GA: gibberellic acid
GA20ox: gibberellin-20 oxidase
GA3ox: gibberellin-3 oxidase
GA2ox: gibberellin-2 oxidase
NCED: 9-*cis*-epoxycarotenoid dioxygenase
ROS: reactive oxygen species
